# The sandfly *Lutzomyia longipalpis* LL5 embryonic cell line has active Toll and Imd pathways and shows immune responses to bacteria, yeast and *Leishmania*

**DOI:** 10.1186/s13071-016-1507-4

**Published:** 2016-04-20

**Authors:** Bruno Tinoco-Nunes, Erich Loza Telleria, Monique da Silva-Neves, Christiane Marques, Daisy Aline Azevedo-Brito, André Nóbrega Pitaluga, Yara Maria Traub-Csekö

**Affiliations:** Laboratório de Biologia Molecular de Parasitas e Vetores, Instituto Oswaldo Cruz - Fiocruz, Av. Brasil 4365, 21045-900 Rio de Janeiro, RJ Brazil

**Keywords:** *Lutzomyia longipalpis*, Embryonic cell line LL5, Innate immune response, Toll pathway, Imd pathway, Antimicrobial peptides

## Abstract

**Background:**

*Lutzomyia longipalpis* is the main vector of visceral leishmaniasis in Latin America. Sandfly immune responses are poorly understood. In previous work we showed that these vector insects respond to bacterial infections by modulating a defensin gene expression and activate the Imd pathway in response to *Leishmania* infection. Aspects of innate immune pathways in insects (including mosquito vectors of human diseases) have been revealed by studying insect cell lines, and we have previously demonstrated antiviral responses in the *L. longipalpis* embryonic cell line LL5.

**Methods:**

The expression patterns of antimicrobial peptides (AMPs) and transcription factors were evaluated after silencing the repressors of the Toll pathway (cactus) and Imd pathway (caspar). AMPs and transcription factor expression patterns were also evaluated after challenge with heat-killed bacteria, heat-killed yeast, or live *Leishmania*.

**Results:**

These studies showed that LL5 cells have active Toll and Imd pathways, since they displayed an increased expression of AMP genes following silencing of the repressors cactus and caspar, respectively. These pathways were also activated by challenges with bacteria, yeast and *Leishmania infantum chagasi*.

**Conclusions:**

We demonstrated that *L. longipalpis* LL5 embryonic cells respond to immune stimuli and are therefore a good model to study the immunological pathways of this important vector of leishmaniasis.

## Background

Previous reports have demonstrated the value of insect cell lines to study complex immune responses. For example, *Drosophila* S2 cells were used to show the independent and synergistic activity of two separate systemic immune pathways [1]. Cell lines of the insect vectors *Aedes albopictus* and *Aedes aegypti* have also been shown to express various immune effector molecules, including antimicrobial peptides (AMPs) [2]. The anopheline mosquitoes *Anopheles gambiae* and *Anopheles stephensi* cell lines Sua1B and MSQ43 have also shown to be immune competent, although key differences were observed in the pathways activated in the cells of these two mosquito species [3]. More recently Aag-2 cells (*Aedes aegypti*) were used to demonstrate immune responses to bacterial and viral challenges [4].

There are two established *Lutzomyia longipalpis* cell lines derived from embryonic tissues, LL5 [5] and Lulo [6]. The Lulo cell line can be infected by *Leishmania infantum chagasi* [7]. Other *Leishmania* species were also able to adhere to Lulo cells at different rates [8].

We previously demonstrated the immune competence for viral infections in LL5 cell line. When LL5 cells were transfected with any double stranded RNA they developed a nonspecific antiviral response [9] reminiscent of an interferon response in mammals.

*L. longipalpis* is the main vector of visceral leishmaniasis in Latin America [10, 11] and can also transmit bacterial and viral diseases [12, 13]. We have previously studied various molecular aspects of *L. longipalpis* immunity and have demonstrated a role for the Imd pathway in modulating vector infection by *Leishmania* [14]. In addition, we showed that defensin gene expression by *L. longipalpis* was modulated by infection with different bacterial species or *Leishmania*, and that the modulation was also influenced by the route of infection [15].

The Imd and Toll pathways are key modulators of innate immunity in insects. After the recognition of pathogen-associated molecular patterns (PAMPs), a signaling cascade is activated leading to the nuclear translocation of NF-ĸB transcriptional factor, initiating the immune response [16]. In *Drosophila melanogaster* DIF and dorsal are NF-ĸB homologue factors involved in the activation of the Toll pathway. The Imd pathway activation leads to nuclear translocation of a different NF-ĸB factor, relish, initiating the immune response through the activation of Imd-related effectors molecules [17, 18].

Here we demonstrate the involvement of the Imd and Toll pathways in immune responses of LL5 cells by revealing the up-regulation of innate immune factors following the silencing of the negative regulators of the Imd and Toll pathways, cactus and caspar, respectively. We also characterized the LL5 cells immunity responses to Gram + and Gram- bacteria, yeast and *Leishmania* challenges.

## Methods

### Cell growth

*Leishmania longipalpis* embryonic LL5 cells were grown in L-15 medium (SIGMA - Aldrich) supplemented with 10 % fetal bovine serum (Laborclin), 10 % tryptose and 1 % penicillin/streptomycin (100 U/ml/100 mg/ml), at 29 °C [9].

Bacteria were grown overnight in LB and yeast in YPAD liquid medium. *Escherichia coli* (strain DH5α) and *Staphylococcus aureus* (strain ATCC 23235) were grown at 37 °C, *Serratia marcescens* isolated from field insects [19] and *Saccharomyces cerevisiae* (strain YRG-2) were incubated at 30 °C. Each culture was pelleted, washed with sterile phosphate-buffered saline (PBS) pH 7.4, resuspended in fresh PBS at OD_600_ = 0.5, immediately autoclaved, cooled to room temperature, and used in challenge procedures of LL5 cells.

*Leishmania infantum chagasi* (MHOM/BR/1974/PP75) obtained from the *Leishmania* collection of Instituto Oswaldo Cruz was maintained in M199 medium, pH 7.0, supplemented with 10 % fetal bovine serum and collected at exponential growth phase, washed with PBS, and resuspended in fresh PBS at 10^7^ parasites/ml for direct use in challenge procedures of LL5 cells.

### LL5 challenges

Cells were maintained in freshly supplemented L-15 medium, seeded in 24 well flat bottom plates. After overnight growth new supplemented L-15 medium was added with the various challenges with microbe/cell ratio of 10 to 1. Non-challenged LL5 cells were used as control. Samples were collected at 2 h, 6 h, 12 h, 24 h, and 48 h post-challenge from two independent experiments by discharging the supernatant medium, washing the cells twice with PBS, adding 1 ml of TRIzol (Invitrogen), and storing at -80 °C for future RNA extraction.

### Double stranded RNA synthesis

dsRNAs were synthesized as described in [9]. Briefly, two rounds of PCR were used for dsRNA in vitro synthesis. The first round the primers were specific for the gene of interest containing adaptors on the 5′ end. In the second PCR reaction the products of the first PCR were used as templates and primers containing the adaptor and T7 promoter sequences. PCR conditions were for the first reaction: 3 min at 95 °C, 35 cycles of 95 °C for 30 s, 57 °C for 45 s and 72 °C for 45 s, followed by 72 °C for 7 min. For the second PCR round, 2 μl of the first reaction were used as template, under the same conditions, and primers containing the adaptor and T7 promoter sequences were used. For in vitro dsRNA transcription, 0.4 μl of PCR-product from the second PCR reaction were used as template, using the MEGAScript T7 kit (Ambion).

All primers used are listed in Table 1. For cactus dsRNA production we used primers DScactus-F and DScactus-R. For caspar dsRNA production we used primers DScaspar-F and DScaspar-R. Accession numbers or description for genes used in primer design are as follows: Cactus (GenBank: EF491250), Attacin (GenBank: KP030755), Cecropin (GenBank: KP030754), Defensin 2 (GenBank: KP030758), Relish (GenBank: KP030757), Dorsal (Vectorbase *L. longipalpis* Scaffold 12). Caspar was described in [14].

**Table 1:** Primers

Name	Sequence
CactusDS-F	5‘ TGGCGCCCCTAGATGCGGTGATTCGGGCTTTAT 3‘
CactusDS-R	5‘ TGGCGCCCCTAGATGGCAGGGGTAGGGATTCATT 3‘
CactusRT-F	5‘ CTAATCCGAATGAATCCCTACCC 3‘
CactusRT-R	5‘ GACCCACGATCACGGCTAGA 3‘
CasparDS-F	5‘ TGGCGCCCCTAGATGAACCCAGTGGTGATTTCCTCG 3‘
CasparDS-R	5‘ TGGCGCCCCTAGATGATAGCGTTTCATCTGCATCCATC 3‘
CasparRT-F	5‘ CCAAAGAGGAGGCAAGAAAGA 3‘
CasparRT-R	5‘ TTCCGCTTCAAGACGCATA 3‘
LucDS-F	5‘ TCCATTCGGTTGGCAGA 3‘
LucDS-R	5‘ CCGTGATGGAATGGAACA 3‘
Attacin-F	5‘ AGGCTGATCCTCTGGGTCCTGT 3‘
Attacin-R	5‘ ATGGGCATGGCAGCGTCTCT 3‘
Cecropin-F	5‘ TGGCAGTCCTGACCACTGGA 3‘
Cecropin-R	5‘ CTTCTCCACTGAACGGTGAACG 3‘
Defensin-2-F	5‘ ATCCATCCTTTATGCAACCG 3‘
Defensin-2-R	5‘ GCCTTTGAGTCGCAGTATCC 3‘
Dorsal-F	5‘ CAATCTCGTGGGAAAGGATG 3‘
Dorsal-R	5‘ ACCCGGAGAGCTTCTTCAAT 3‘
Relish-F	5‘ ACGGGATTGCTCTGACTACG 3‘
Relish-R	5‘ ACGGCTTGTAGGTGAAGTGC 3‘
RP49-F	5‘ GACCGATATGCCAAGCTAAAGCA 3‘
RP49-R	5‘ GGGGAGCATGTGGCGTGTCTT 3‘
T7-Primer	5‘ CCGTAATACGACTCACTATAGGGTGGCGCCCCTAGATG 3‘

### LL5 transfection

Transfection was performed using the lipid reagent DharmaFECT1 (Thermo Scientific). A mixture was prepared containing 0.25 ml DharmaFECT, 23.25 ml DCCM (DHARMACON) and 1.5 ml dsRNA for a final concentration of 30 nM. LL5 cells were maintained in this mixture for 16 h and then incubated for 12, 24 and 48 h at 30 °C, before being resuspended in TRIzol (Ambion).

### RNA and cDNA preparation, and qPCR

RNA was prepared from LL5 cells using TRIzol following manufacturer’s instructions and the RNA was treated with RQ1 RNase-Free DNase (Promega). cDNA was synthetized with the kit SuperScript III First-Strand Synthesis System for RT-PCR (Invitrogen) and oligo dT_(16)_ primer. Quantitative PCR (qPCR) was performed using the kit iQTM SYBR Green Supermix (Applied Biosystems). All experiments were performed using two biological replicates. Expression was normalized using the constitutive gene rp49 [20] and relative levels of RNA expressed were calculated using the ΔΔCT method [21]. Primers for cecropin, defensin, attacin and relish are described in Table 1.

Significant differences in gene expression were determined by the Mann-Whitney or ANOVA tests with multiple comparisons of Games-Howell or Tukey. All tests were performed with reliable level of 95 % (α = 0.05). The statistical analyses were performed using the GraphPad Prism 5 software (GraphPad Software, Inc.).

## Results

### Silencing of the toll pathway repressor cactus affects AMP genes expression

**Fig. 1:**
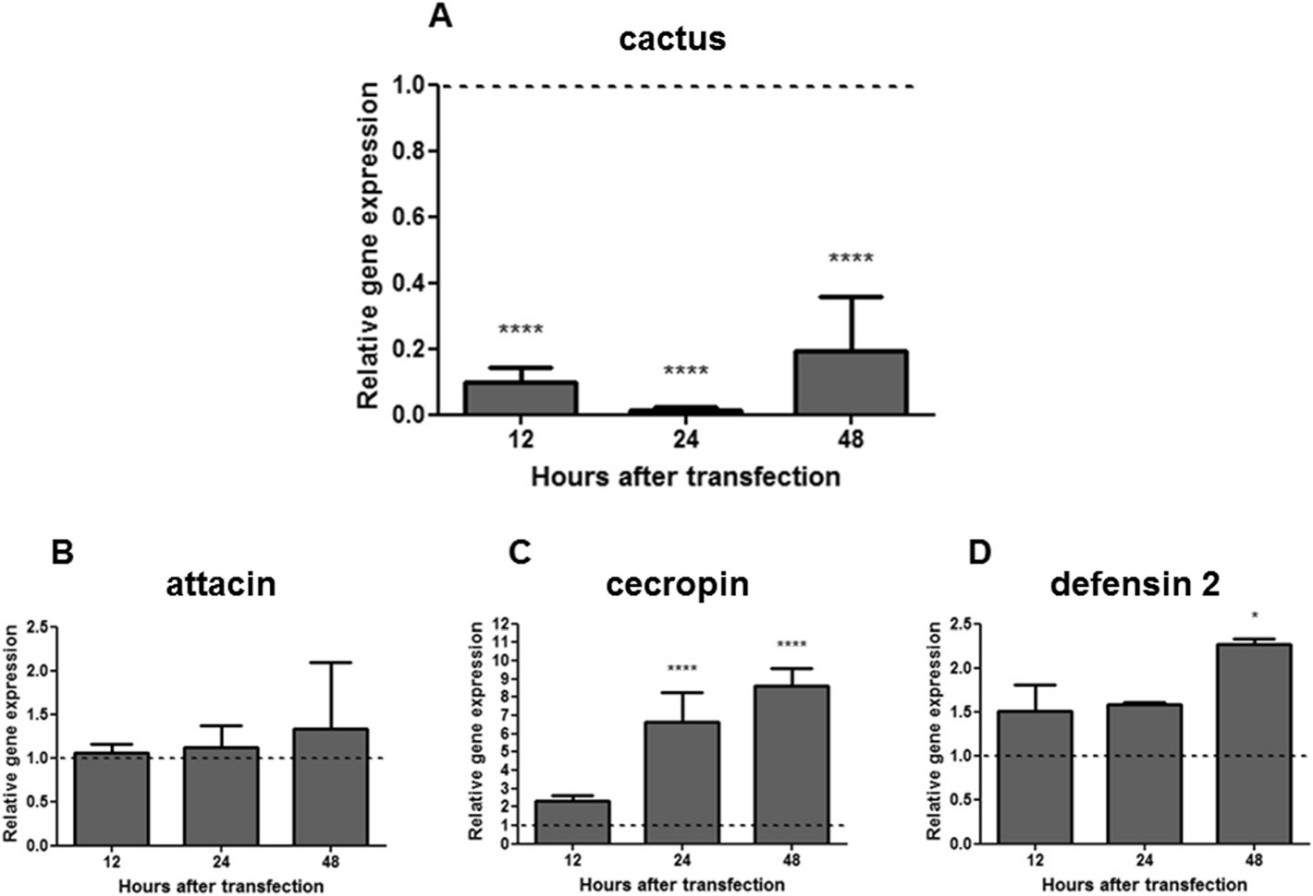
Cactus silencing and relative gene expression of the AMPs attacin, cecropin and defensin 2 determined by qPCR: **a** Gene expression of the Toll pathway repressor cactus in LL5 cells transfected with cactus dsRNA; **b**, **c** and **d** Gene expression of the AMPs attacin, cecropin and defensin 2 in LL5 cells after cactus silencing. Quantifications were normalized relative to the housekeeping gene rp49, and relative gene expression expressed as fold change calculated relative to mock transfected control group. Dotted line indicates the gene expression of control transfected with luciferase dsRNA. Bars represent mean with standard error (SEM) of two biological replicates from independent experiments collected at 12, 24 and 48 h post dsRNA transfection. ANOVA test with multiple comparisons of Games-Howell or Tukey was used. Bonferroni correction was used in the analyses of the samples. *P*-values: *P < 0.05; ****P < 0.0001

The role of the Toll pathway on AMP production was evaluated by examining the effect of silencing the Toll repressor cactus with double-stranded RNA (dsRNA) for the cactus gene. The silencing of cactus was evaluated by qPCR and shown to be very effective up to 48 h post-transfection (Fig. 1a). The silencing of cactus had no effect on attacin expression (Fig. 1b) but led to the increased expression of cecropin (Fig. 1c) and defensin 2 (Fig. 1d), 24 and 48 h post-transfection.

### Silencing of the Imd pathway repressor caspar affects the expression of AMP genes and the transcription factor relish

**Fig. 2:**
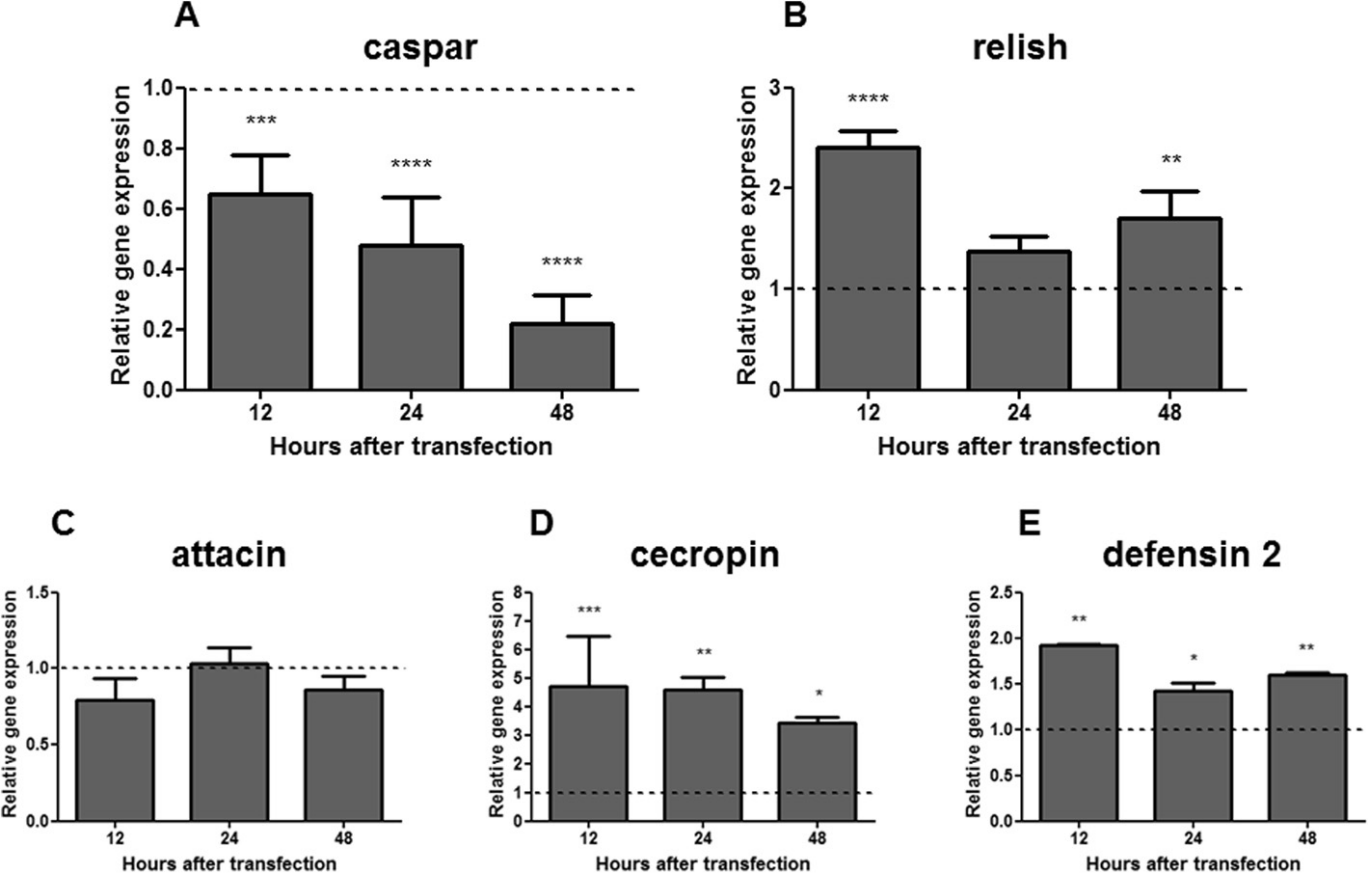
Caspar silencing and relative gene expression of the AMPs attacin, cecropin, defensin 2 and transcription factor relish determined by qPCR: **a** Gene expression of the Imd pathway repressor caspar in embryonic LL5 cells transfected with caspar dsRNA; **b**, **c**, **d** and **e** Gene expression of the AMPs attacin, cecropin and defensin 2, and the transcription factor relish in LL5 after caspar silencing. Quantifications were normalized relative to the housekeeping gene rp49, and relative gene expression expressed as fold change calculated relative to unchallenged control group. Dotted line indicates the gene expression of controls transfected with luciferase dsRNA. Bars represent the SEM of two biological replicates from independent experiments collected at 12, 24 and 48 h post dsRNA transfection. ANOVA test with multiple comparisons of Games-Howell or Tukey was used. Bonferroni correction was used in the analyses of the samples. *P*-values: *P < 0.05; **P < 0.01; ***P < 0.001; ****P < 0.0001

The role of the Imd pathway on LL5 cells AMPs production was evaluated by examining the effect of caspar silencing with caspar dsRNA. After dsRNA transfection, caspar was silenced effectively for up to 48 h (Fig. 2a). Consistent with the expected role of caspar in the Imd pathway activation, the transcription factor relish showed an increased expression with caspar dsRNA transfection (Fig. 2b). Similar to the situation with cactus silencing, caspar silencing had no effect on attacin expression (Fig. 2c) but caused increased expression levels of cecropin and defensin 2 (Fig. 2d and e).

### Immune responses of LL5 cells exposed to bacteria or yeast challenge

The role of the Toll and Imd pathways in LL5 cell responses to extracts from Gram + and Gram- bacteria, and yeast, were evaluated. We assessed the relative gene expression of the repressors cactus and caspar, the transcription factors dorsal and relish, and the AMPs attacin, cecropin, and defensin 2 post-challenge (PC) using qPCR.

**Fig. 3:**
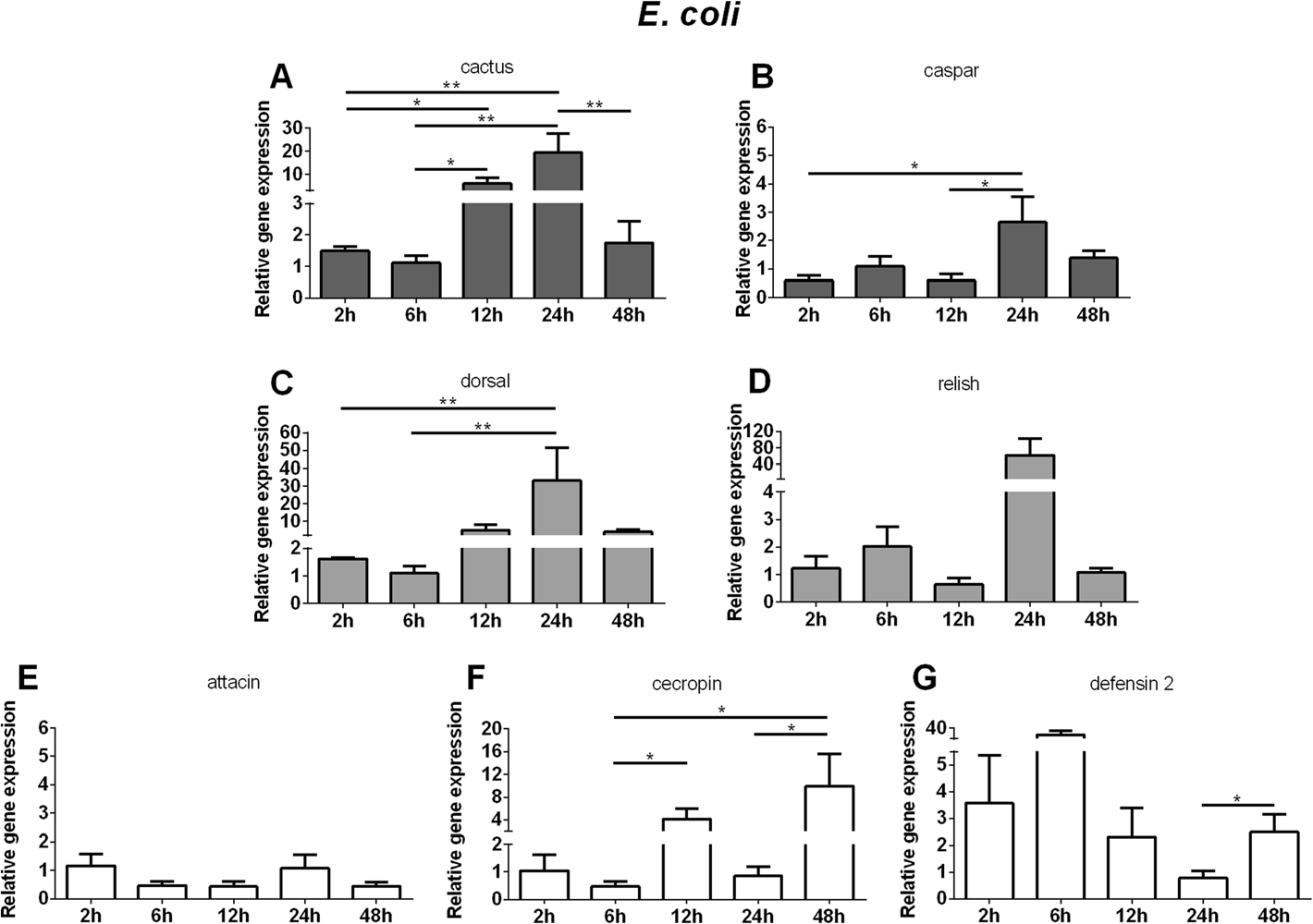
Relative gene expression of the immune pathways repressors, transcription factors and AMPs determined by qPCR after *E. coli* challenge: **a** and **c** Relative gene expression of cactus and dorsal regulators of Toll pathway; **b** and **d** Relative gene expression of caspar and relish regulators of Imd pathway; **e**, **f**, and **g** Relative gene expression of AMPs attacin, cecropin, and defensin 2. Bars represent mean with standard error (SEM) of two biological replicates from independent experiments collected at 2 h, 6 h, 12 h, 24 h, and 48 h post-challenge. Quantifications were normalized relative to the housekeeping gene rp49, and relative gene expression expressed as fold change calculated relative to unchallenged control group. Mann-Whitney test was used to verify significant differences. *P*-values: *P < 0.05; **P < 0.01

After LL5 exposure to *E. coli*, cactus expression increased from 12 to 24 h PC (Fig. 3a), while caspar expression presented an increase after 24 h (Fig. 3b). Dorsal expression increased from 12 to 24 h PC (Fig. 3c) and relish presented a non-significant increase at 24 h PC (Fig. 3d). The AMP attacin showed no modulation (Fig. 3e) while cecropin increased expression at 12 and 48 h (Fig. 3f). Defensin 2 presented a tendency towards increased expression at 2 to 6 h, which was reduced at 24 h to control levels, increasing again at 48 h PC (Fig. 3g).

**Fig. 4:**
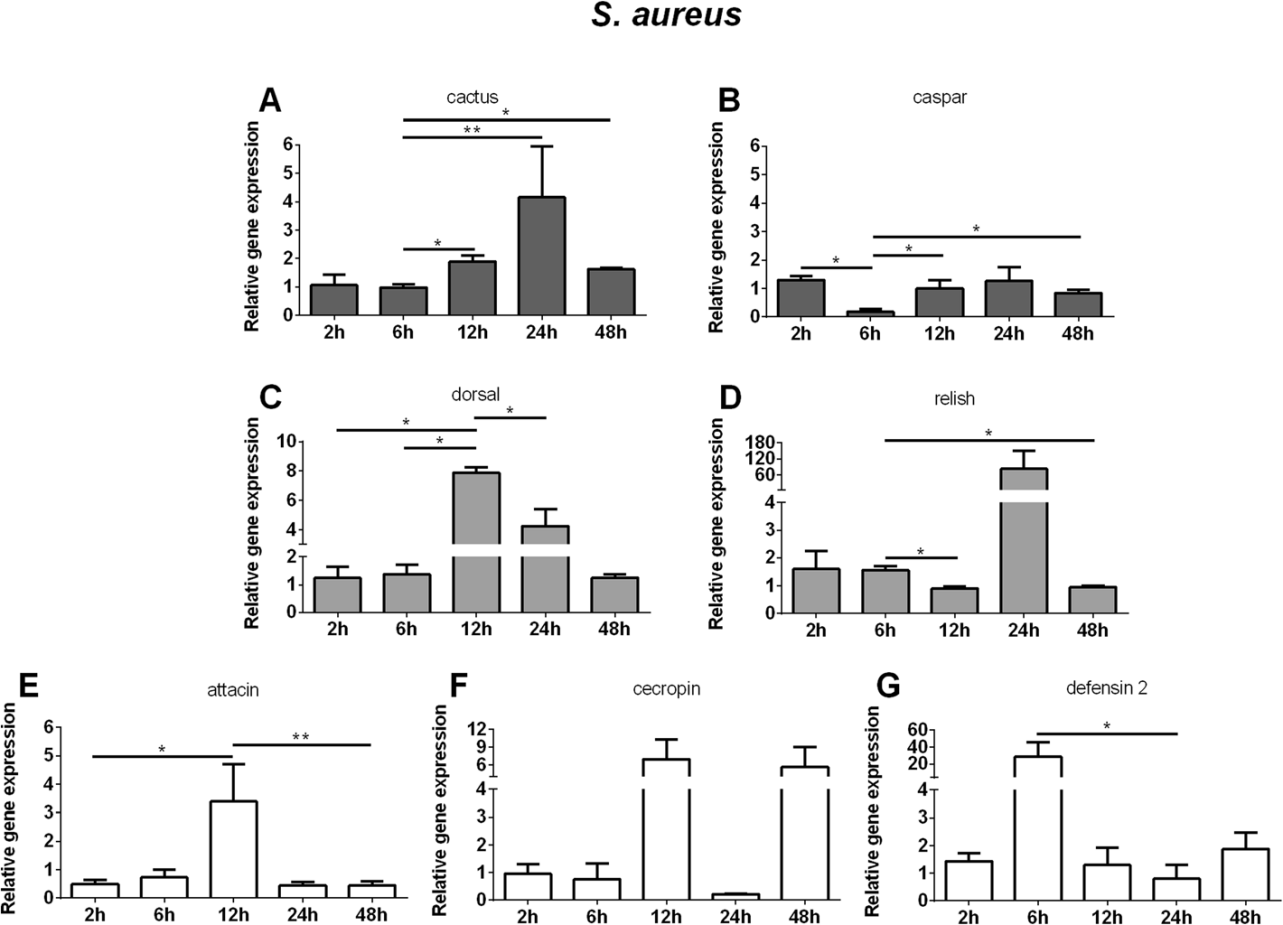
Relative gene expression of the immune pathways repressors, transcription factors and AMPs determined by qPCR after *S. aureus* challenge: **a** and **c** Relative gene expression of cactus and dorsal regulators of Toll pathway; **b** and **d** Relative gene expression of caspar and relish regulators of Imd pathway; **e**, **f**, and **g** Relative gene expression of AMPs attacin, cecropin, and defensin 2. Bars represent mean with standard error (SEM) of two biological replicates from independent experiments collected at 2 h, 6 h, 12 h, 24 h, and 48 h post-challenge. Quantifications were normalized relative to the housekeeping gene rp49, and relative gene expression expressed as fold change calculated relative to unchallenged control group. Mann-Whitney test was used to verify significant differences. *P*-values: *P < 0.05; **P < 0.01

Upon *S. aureus* challenge, LL5 cells demonstrated an up-regulation of cactus expression from 12 h to 24 h PC (Fig. 4a) and caspar an overall constant expression which decreased at 6 h (Fig. 4b). Dorsal expression increased from 12 h to 24 h PC (Fig. 4c) while relish expression increased initially at 2 to 6 h PC, and decreased at 12 h (Fig. 4d). Defensin 2 presented an increased expression at 6 h PC (Fig. 4g) while attacin increased at 12 h PC (Fig. 4e) and cecropin had a non-significant expression increase at 12 and 48 h (Fig. 4f).

**Fig. 5:**
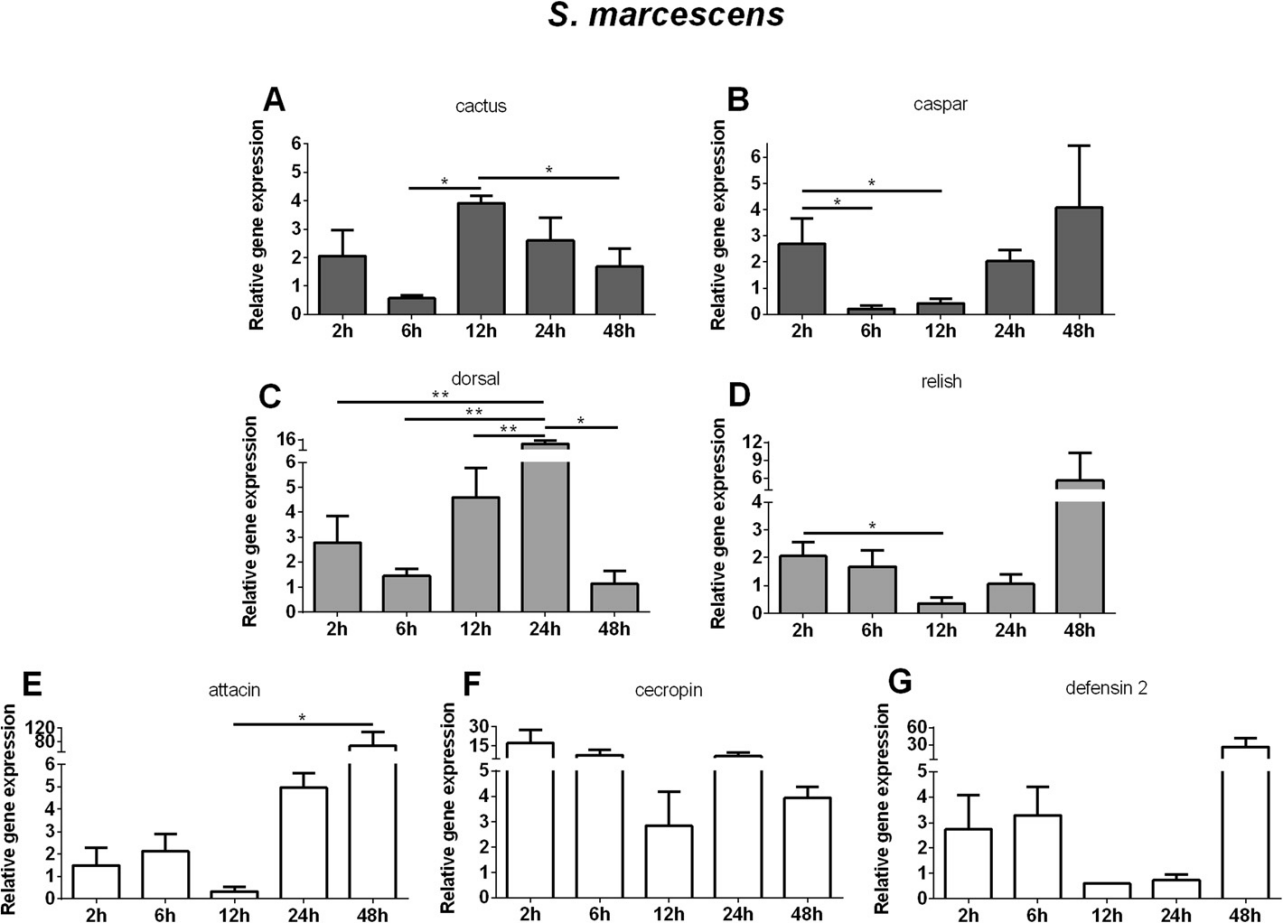
Relative gene expression of the immune pathways repressors, transcription factors and AMPs determined by qPCR after *S. marcescens* challenge: **a** and **c** Relative gene expression of cactus and dorsal regulators of Toll pathway; **b** and **d** Relative gene expression of caspar and relish regulators of Imd pathway; **e**, **f**, and **g** Relative gene expression of AMPs attacin, cecropin, and defensin 2. Bars represent mean with standard error (SEM) of two biological replicates from independent experiments collected at 2 h, 6 h, 12 h, 24 h, and 48 h post-challenge. Quantifications were normalized relative to the housekeeping gene rp49, and relative gene expression expressed as fold change calculated relative to unchallenged control group. Mann-Whitney test was used to verify significant differences. *P*-values: *P < 0.05; **P < 0.01

When LL5 cells were challenged with *S. marcescens*, cactus expression was up-regulated at 12 h PC (Fig. 5a) and caspar showed an initial increase of expression at 2 h followed by reduction at 6 and 12 h (Fig. 5b). Dorsal expression increased from 12 to 24 h PC (Fig. 5c) and relish presented an initial increase at 2 to 6 h, and decrease at 12 h (Fig. 5d). Attacin presented increased expression at 48 h (Fig. 5e). Cecropin presented rather constant levels of expression (Fig. 5f) and defensin 2 presented a tendency of increase from 2 h to 6 h and at 48 h (Fig. 5g).

**Fig. 6:**
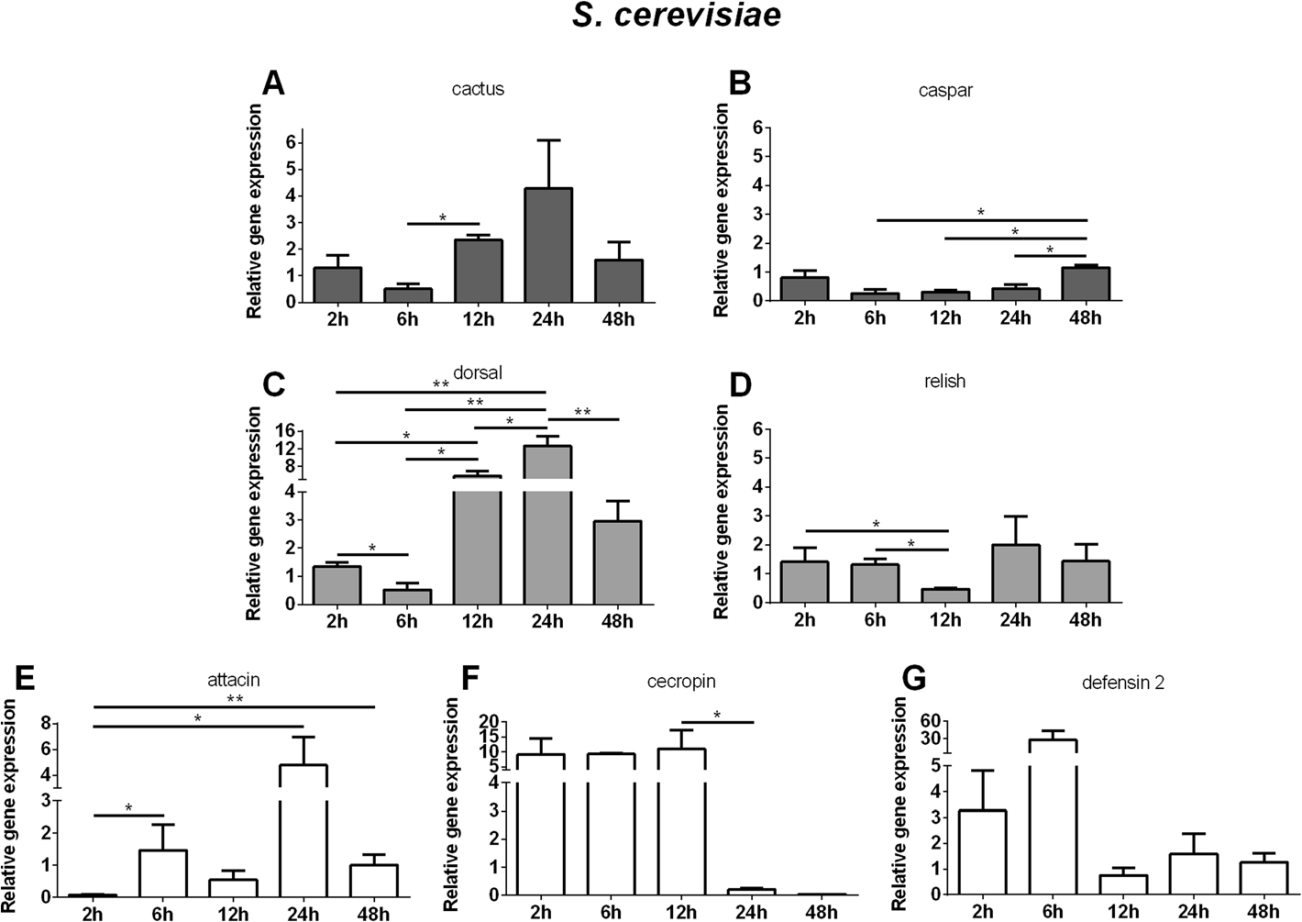
Relative gene expression of the immune pathways repressors, transcription factors and AMPs determined by qPCR after *S. cerevisiae* challenge: **a** and **c** Relative gene expression of cactus and dorsal regulators of Toll pathway. **b** and **d** Relative gene expression of caspar and relish regulators of Imd pathway. **e**, **f**, and **g** Relative gene expression of AMPs attacin, cecropin, and defensin 2. Bars represent mean with standard error (SEM) of two biological replicates collected at 2 h, 6 h, 12 h, 24 h, and 48 h post-challenge. Quantifications were normalized relative to the housekeeping gene rp49, and relative gene expression expressed as fold change calculated relative to unchallenged control group. Mann-Whitney test was used to verify significant differences. *P*-values: * *P* < 0.05; **P < 0.01

When LL5 were exposed to *S. cerevisiae* an up-modulation of cactus was seen from 12 to 24 h PC (Fig. 6a) and caspar down modulation from 6 to 24 h PC (Fig. 6b). Dorsal expression increased from 12 h to 24 h PC (Fig. 6c) and relish had an initial increase at 2 to 6 h and decrease at 12 h PC (Fig. 6d). Defensin 2 presented a non significant up-regulation at 2 and 6 h PC (Fig. 6g) and attacin a significant increase at 24 h (Fig. 6e). Cecropin expression showed an early increase from 2 to 12 h (Fig. 6f).

### LL5 immune responses to *Leishmania* challenge

**Fig. 7:**
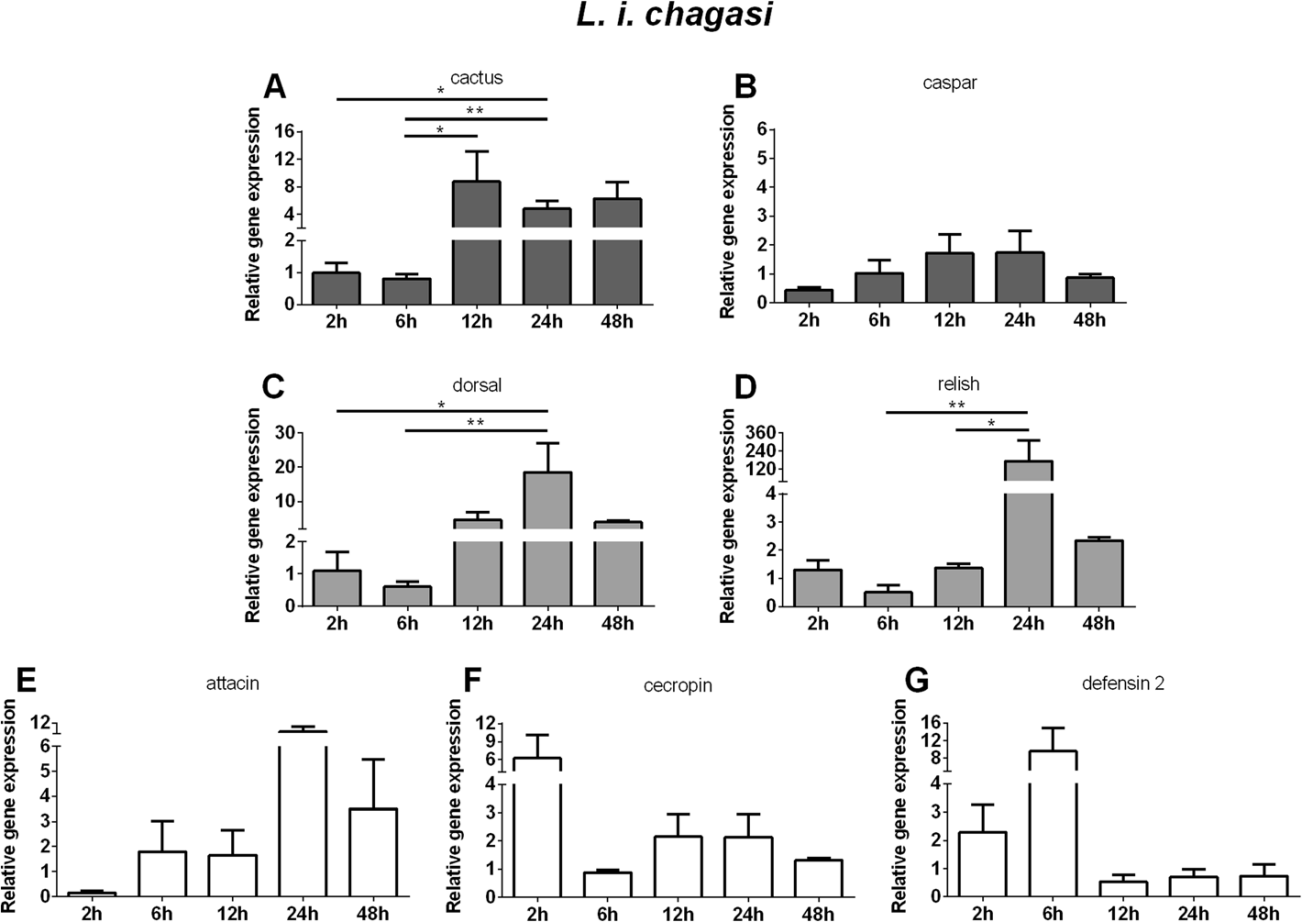
Relative gene expression of the immune pathways repressors, transcription factors and AMPs determined by qPCR after *L. i. chagasi* challenge: **a** and **c** Relative gene expression of cactus and dorsal regulators of Toll pathway. **b** and **d** Relative gene expression of caspar and relish regulators of Imd pathway. **e**, **f**, and **g** Relative gene expression of AMPs attacin, cecropin, and defensin 2. Bars represent mean with standard error (SEM) of two biological replicates collected at 2 h, 6 h, 12 h, 24 h, and 48 h post-challenge. Quantifications were normalized relative to the housekeeping gene rp49, and relative gene expression expressed as fold change calculated relative to unchallenged control group. Mann-Whitney test was used to verify significant differences. *P*-values: * *P* < 0.05; **P < 0.01

The role of the Toll and Imd pathways in LL5 cells challenged with live *L. i. chagasi* was also evaluated. Cactus, the negative regulator of the Toll pathway, demonstrated an up regulated gene expression at 12 and 24 h PC (Fig. 7a). Caspar, the negative regulator of the Imd pathway presented no significant change (Fig. 7b). The positive regulators dorsal expression increased from 12 to 24 h PC (Fig. 7c) while relish expression increased at 24 h PC (Fig. 7d). The AMP defensin 2 presented a tendency for increased expression at 2 h and 6 h PC (Fig. 7g), attacin at 24 h (Fig. 7e) and cecropin showed tendency for increase at 2 h PC (Fig. 7f).

## Discussion

Our results indicate that LL5 cells are a good model to study immune responses in *L. longipalpis*, an important vector of leishmaniasis and other pathogens. Innate immunity is one of insects’ major defense mechanisms which is quickly activated after a challenge and shows a degree of specificity to different classes of microorganisms [18]. The innate immune system includes recognition of molecules whose engagement drives signaling cascades that control a series of effector mechanisms that can resolve infections. Insects can use both humoral and cell defenses, the latter involving hemocytes present in the hemolymph [22]. Insects possess the three main immune pathways also found in other organisms, Toll, Imd and Jak/STAT. The most studied regulatory molecules are cactus and dorsal, the repressor and transcription factors of the Toll pathway, and caspar and relish, the repressor and transcription factors of the Imd pathway. The production of AMPs such as attacin, cecropin, defensin and other effector molecules occurs when transcription factors are translocated to the cell nucleus. Although redundancy among the pathways is known to exist, Gram + bacteria and fungi are thought to preferentially stimulate the Toll pathway, whereas Gram- bacteria are thought to preferentially activate the Imd pathway [23–25]. Most of the knowledge on insect immunity has originated from studies of *Drosophila* and mosquito vectors. In other vectors, such as sandflies, there is a gap regarding the understanding of immune responses. For instance, there is no information regarding which transcription factors and AMPs are involved in the Toll and Imd pathways.

The data present in this work performed on *L. longipalpis* LL5 cells suggest the redundant regulation for the production of cecropin and defensin 2 through the Toll and Imd pathways. This is not unexpected, since in general all pathways (Toll, Imd and Jak/STAT) share some common target genes [26]. Indeed, the *L. longipalpis* defensin 1 gene has several potential binding sites for immune related transcription factors in the 5′UTR region, including dorsal [15]. In MSQ43 *A. stephensi* cells cecropin and defensin are also regulated by the Toll and Imd pathways [3], reinforcing the fact that some AMPs are not regulated in an exclusive way in some insects. Our results show that expression of the attacin gene was not modulated by silencing the inhibitors of the Toll and Imd pathways, although cactus is described in the literature as a negative regulator of the Toll pathway that regulates attacin expression both in *Drosophila* and *Anopheles* [27]. Thus, it appears that this AMP may be regulated by an alternative pathway.

The use of live bacteria and yeast in co-culture challenges limits the interaction assays to short time periods due to the fast growing rate of the most microorganisms compared to the cell line being tested. To overcome this limitation we utilized heat-killed microbes. The use of these preparations might change their PAMP profile relative to live microbes, but heat-killed microbes are commonly used to challenge insect cell lines [4, 28] and live insects [29] to study immune phenotypes. Additionally, some studies have shown increased immunogenicity induced by heat-killed microbes possibly because of changes in PAMP exposure. For example, a heat-killed *Salmonella enterica* induced higher production of reactive oxygen species (ROS) in tobacco BY-2 cells [30]. In our experiments we used autoclaved bacteria or yeast in order to follow the effect of the challenges over two days. Autoclaved *E. coli*, *S. aureus*, *S. marcescens*, and *S. cerevisiae* caused cactus and dorsal to increase expression at 12 h and 24 h indicating that the Toll pathway was both activated and inhibited after these challenges. The synchronicity of expression may be explained by the fact that cactus remains bound to dorsal until release and translocation to the nucleus [31]. Also, as seen in organisms ranging from mammals to *Drosophila* [32] hyperactivated immune responses may be detrimental, and are thus finely modulated. Similarly, *A. aegypti* Aag-2 cells challenged with Gram + and Gram- bacteria, and yeast Zymosan, had increased expression of cactus and serpin indicating that the Toll pathway was activated [4]. The fact that caspar and relish had their expression reduced at 6 h or 12 h and then increased at 24 h or 48 h PC indicates that the Imd pathway was also activated upon challenges. In the case of *S. marcescens*, an insect pathogen, caspar and relish were up-regulated as early as 2 h, suggesting that the Imd pathway may be activated more rapidly by this pathogen.

The expression of AMPs after these challenges varied considerably and in some cases, although the expression had a tendency to increase, there was no statistical significance. Attacin was up regulated after *S. aureus*, *S. cerevisiae* and *S. marcescens* challenge concomitantly to dorsal up-regulation, suggesting that attacin expression may be regulated by dorsal. Nevertheless, since attacin was not altered after cactus or caspar silencing, we may consider that dorsal may be regulated in a more complex manner in LL5 cells. Cecropin was increased after *E. coli*, *S. aureus*, *S. marcescens and S. cerevisiae* challenges, in a closer synchrony to relish modulation, suggesting that cecropin might be under the regulation of the Imd pathway. In *Drosophila* S2 cells, attacinA and cecropinA1 were induced after challenge with *E. coli* peptidoglycan and were associated with the Imd pathway activation [1], indicating once again the peculiarities of different insects and/or insect cell lines. Defensin 2 expression varied following different challenges, but in the case of *S. marcescens*, defensin 2 seemed to be regulated by the Imd pathway. In *A. aegypti* Aag-2 cells, defensin was up-regulated 24 h after exposure to the Gram + *Micrococcus luteus*, Gram- *Enterobacter cloacae* or Zymosan, after activation of the Toll and Imd pathways [4]. We cannot exclude other AMPs not investigated in this work that may be produced in response to these microbial challenges and may influence the balance of the cell immune response. In sandfly adult females there is at least one other defensin that has been described and shown to be expressed in response to bacteria [15, 33].

We used pathogenic and non-pathogenic microorganisms in our challenges. *E. coli* (DH5α strain) and *S. cerevisiae* (YRG-2 strain) are non-pathogenic. Although *S. aureus* can produce enterotoxins [34] both *S. aureus* and *E. coli* were found in normal *L. longipalpis* microbiota [35, 36]. *S. marcescens* found in field insects [35, 37] and laboratory strains may be pathogenic to *L. longipalpis* [38].

We challenged LL5 cells with live *L. i. chagasi* parasites. This model of *Leishmania*-embryonic cell interaction in co-culture has previously been employed with Lulo cells. In those studies, *L. i. chagasi* (WR strain) promastigotes were viable and able to multiply during initial days of the interaction with the Lulo cells, but the cells rounded up and detached from the culture flask bottom after 5 days of contact with the parasites [6]. Thus, this interaction posed a severe challenge to the insect cell line. An alternative *L. i.chagasi* isolate (MH/CO/84/Cl-044B) when in contact with Lulo cells showed attachment at 3 h, reaching the strongest attachment at 12 h, and after day 2 parasites were detected internalized in the parasitophorous vacuoles [7].

Our results show that exposure to *L. i. chagasi* increased cactus and dorsal expression in LL5 cells from 12 h to 48 h post-challenge, indicating that Toll pathway was activated. Accordingly, *Leishmania* was found to be strongly attached to Lulo cells at 12 h after interaction [7]. We observed that relish was increased at 24 h after *Leishmania* challenge indicating that this transcription factor was also expressed in response to *Leishmania* challenge. If a parallel is traced to Lulo cell experiments [7], at 24 h post interaction parasites were in the process of internalization. In LL5 cells we did not detect a significant alternation of caspar expression after *Leishmania* challenge, whereas previous reports in *L. longipalpis* adult females, showed caspar expression decreased at 72 h, at time point when the parasites were expected to be directly interacting with the insect gut [14]. Furthermore, the activation of the Imd pathway through the silencing of caspar caused a reduction on *Leishmania* survival [14]. The expression of AMPs upon *Leishmania* interaction with LL5 cells in our studies resulted in highly variable changes in expression. Specifically, cecropin expression reached its peak at 2 h, defensin 2 reached its peak at 6 h, and attacin expression did not peak until 24 h. An interesting observation is that early responsive AMPs cecropin and defensin 2 were tightly negatively regulated by cactus consistent with being under control of the Toll pathway. On the other hand, the late responsive AMP attacin seemed to be regulated by relish with no influence of caspar, probably being regulated in a more complex manner. The expression of only one AMP (defensin) has been reported in the context of *Leishmania*-challenged insects [15, 33], and there have been no reports of AMP regulation in *Leishmania*-challenged embryonic sandfly cell lines.

**Fig. 8:**
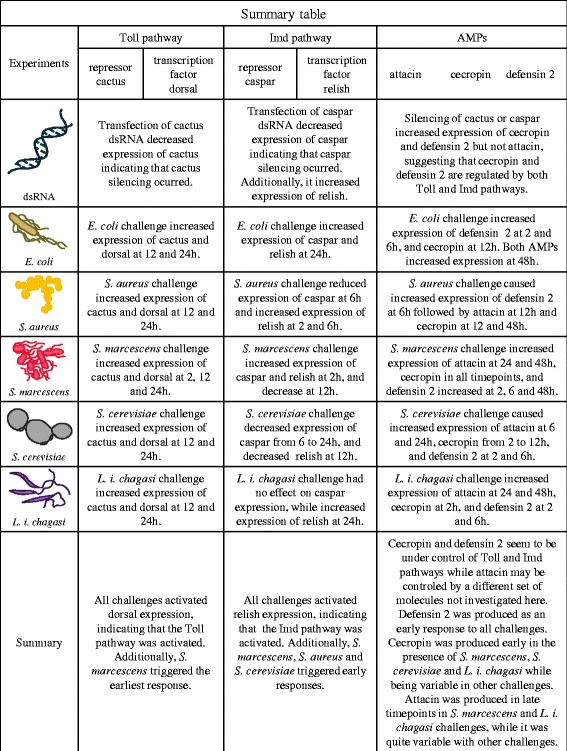
Summary Table

In summary, *S. marcescens* challenge was capable of triggering an early response indicated by the expression levels of dorsal and relish. An early induction of relish is also observed after *S. aureus and S. cerevisiae* challenges. Later induction of both transcription factors were also observed in all challenges. Regarding the AMPS, defensin 2 was readily expressed upon all challenges, cecropin was also readily expressed upon *S. marcescens*, *S. cerevisiae* and *L. i. chagasi* challenges, while attacin is expressed later in *S. aureus, S. marcescens*, *S. cerevisiae* and *L. i. chagasi* but not by *E. coli* challenge. For clarity our results are summarized in Fig. 8.

## Conclusion

The difficulty of working with live insects, which is pronounced in the case of sandflies due to their small size and maintenance limitations, makes insect cell lines valuable models for the study of the molecular basis of insect immunity. *Drosophila* cell lines have been used extensively to study a broad range of aspects of insect physiology, and much of the work done with these cell lines has been aided by gene silencing methods. Here we have established the utility and feasibility of using the easily maintained LL5 *L. longipalpis* embryonic cell line for unraveling aspects of the immunity of these vectors. These cells not only possess active Toll and Imd pathways, they also react in specific ways to different biological stimuli in ways concordant with available information in other systems. We thus conclude that the LL5 cell line is a good model for studying sandfly immunity.
